# mFast‐SeqS‐based aneuploidy score in circulating cell‐free DNA is a prognostic biomarker in prostate cancer

**DOI:** 10.1002/1878-0261.13449

**Published:** 2023-08-18

**Authors:** Khrystany T. Isebia, Bianca Mostert, Teoman Deger, Jaco Kraan, Vanja de Weerd, Esther Oomen‐de Hoop, Paul Hamberg, Brigitte C. M. Haberkorn, Helgi H. Helgason, Ronald de Wit, Ron H. J. Mathijssen, Martijn P. Lolkema, Saskia M. Wilting, Job van Riet, John W. M. Martens

**Affiliations:** ^1^ Department of Medical Oncology, Erasmus MC Cancer Institute University Medical Center Rotterdam The Netherlands; ^2^ Department of Internal Medicine Franciscus Gasthuis & Vlietland Rotterdam/Schiedam The Netherlands; ^3^ Department of Medical Oncology Maasstad Hospital Rotterdam The Netherlands; ^4^ Department of Medical Oncology Haaglanden Medical Centre The Hague The Netherlands

**Keywords:** aneuploidy scores, ctDNA, metastasis, mFast‐SeqS, next generation sequencing, prostate cancer

## Abstract

Multiple prognostic biomarkers, including circulating tumour cell (CTC) counts, exist in metastatic castration‐resistant prostate cancer (mCRPC) patients, but none of them have been implemented into daily clinical care. The modified fast aneuploidy screening test‐sequencing system (mFast‐SeqS), which yields a genome‐wide aneuploidy score, is able to reflect the fraction of cell‐free tumour DNA (ctDNA) within cell‐free DNA (cfDNA) and may be a promising biomarker in mCRPC. In this study, we investigated the prognostic value of dichotomized aneuploidy scores (< 5 vs. ≥ 5) as well as CTC counts (< 5 vs. ≥ 5) in 131 mCRPC patients prior to treatment with cabazitaxel. We validated our findings in an independent cohort of 50 similarly treated mCRPC patients. We observed that, similar to the dichotomized CTC count [HR: 2.92; 95% confidence interval (CI);1.84–4.62], dichotomized aneuploidy scores (HR: 3.24; CI: 2.12–4.94) significantly correlated with overall survival in mCRPC patients. We conclude that a dichotomized aneuploidy score from cfDNA is a prognostic marker for survival in mCRPC patients within our discovery cohort and in an independent mCRPC validation cohort. Therefore, this easy and robust minimally‐invasive assay can be readily implemented as a prognostic marker in mCRPC. A dichotomized aneuploidy score might also be used as a stratification factor in clinical studies to account for tumour load.

## Graphical abstract

The dichotomized mFast‐SeqS‐based aneuploidy score from cfDNA is a prognostic marker for survival in mCRPC patients. This easy, robust, low‐cost, and minimally invasive assay can be readily implemented as a prognostic marker in mCRPC. This assay represents an attractive stand‐alone stratification tool for use in daily clinical practice, and could also aid as a stratification factor in clinical studies.

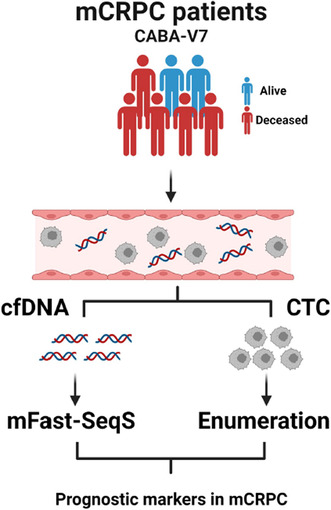

**Abbreviations**
ALB: albumin levels
ANC: absolute neutrophil count
cfDNA: cell‐free DNA
CI: confidence interval
ctDNA: cell‐free tumour DNA
EpCam: epithelial cell adhesion molecule
HBG: hemoglobin levels
HR: hazard ratio
LDH: lactate dehydrogenase levels
mCRPC: metastatic castration‐resistant prostate cancer
mFast‐SeqS: modified Fast Aneuploidy Screening Test‐Sequencing System
NIPT: noninvasive prenatal test
OS: overall survival

## 1 Introduction

The daily clinical care of metastatic castration‐resistant prostate cancer (mCRPC) patients could be improved by utilizing non‐invasively derived prognostic markers. Circulating tumour cells (CTCs) and circulating tumour‐derived DNA (ctDNA) comprise two minimally invasive and safely obtainable biomarkers from liquid biopsies [1]. Extensive efforts have been undertaken to investigate the use of CTCs as prognostic or predictive biomarkers in mCRPC. The potential applications of CTC enumeration are far‐reaching and could also lead to, assist in treatment monitoring and treatment response, and prognosticate on progression‐free survival [2, 3, 4, 5, 6]. The current implementation of predicting overall survival (OS) through CTC enumeration depends on a dichotomized threshold of < 5 or ≥ 5 CTCs in 7.5 mL of blood for prognostication of good versus adverse outcomes in mCRPC patients [5]. The sole FDA‐approved system for this approach is the CellSearch system. However, an important limitation of the CellSearch system remains its dependency on epithelial cell adhesion molecule (EpCAM), thereby making it only capable of capturing EpCAM‐positive CTCs [7]. To overcome this limitation while also possibly increasing sensitivity and reducing costs, ctDNA‐based genotyping has emerged as a promising alternative with potential diagnostic, predictive and prognostic implications. The relatively affordable and robust modified fast aneuploidy screening test‐sequencing system (mFast‐SeqS), which was originally developed to detect foetal aneuploidy within maternal plasma, was recently found capable of estimating tumour fractions within the total pool of cell‐free DNA (cfDNA) [8]. mFast‐SeqS yields a genome‐wide aneuploidy score that is able to reflect the fraction of cell‐free tumour DNA (ctDNA) within cell‐free DNA (cfDNA) by sequencing unique LINE‐1 elements from plasma samples and subsequently mapping them to the human reference genome. Subsequently, sample‐specific Z‐scores per chromosome arm can be determined and summed into a genome wide aneuploidy score per patient. Circulating tumour‐derived aneuploidy is correlated with underlying tumour content and allows for monitoring without prior knowledge of the genetic composition of the tumour. Dichotomization based on aneuploidy scores has already been found valuable for the prognostication of metastatic breast cancer and advanced breast cancer [9, 10, 11]. Since aneuploidy is considered a hallmark of cancer and research has revealed that aneuploidy could even be associated with lethal progression in prostate cancer, the exploitation of dichotomized aneuploidy scores as a prognostic marker for mCRPC patients could represent an attractive alternative or complement to CTC enumeration [12]. From a clinical and molecular perspective, prostate cancer is considered a heterogeneous disease with many routes leading to aneuploidy. Therefore, having a uniform and non‐invasive test to determine aneuploidy based on chromosomal read out is worth investigating [13, 14].

In this manuscript, we investigated the prognostic value of mFast‐SeqS‐derived dichotomized aneuploidy scores for mCRPC patients and compared their performance to dichotomized CTC counts.

## 2 Materials and methods

### 2.1 Patient inclusion and clinical parameters

For our discovery cohort, we included 131 out of 137 mCRPC patients with known CTC counts from the CABA‐V7 trial (MEC16‐703), as previously detailed by Isebia *et al*. [15]. Six patients were excluded due to insufficient available plasma for mFast‐SeqS. In the CABA‐V7 trial, a prospective, multicentre, single arm phase II clinical trial, mCRPC patients were included who progressed after treatment with docetaxel. In this trial, patients were screened for the presence of CTCs and AR‐V7 status. This trial was conducted in accordance with the Declaration of Helsinki and approved by the independent Dutch medical ethical committee (BEBO) (MEC 16‐703). All patients provided written informed consent before any study procedure was performed. The primary aim of this study was to evaluate whether cabazitaxel would be a viable alternative for AR‐V7 positive mCRPC patients.

As an independent validation cohort, we selected mCRPC patients (*n* = 50) with known CTC counts from the CABARESC trial (MEC 11‐324; *n* = 224), as previously detailed by Nieuweboer *et al*. [16]. The CABARESC trial, a randomized, multicentre, phase II, open‐label study, included mCRPC patients with documented disease progression during or after docetaxel treatment. This trial was conducted in accordance with the Declaration of Helsinki and approved by the local institutional review board (METC) (MEC 11‐324). All patients provided written informed consent before any study procedure was performed. The primary aim of this study was to evaluate the effects of budesonide on cabazitaxel pharmacokinetics and cabazitaxel‐induced diarrhoea. This validation series was established to ensure sufficient plasma and comparable baseline clinical characteristics to the discovery cohort. Thereby, we ensured the selection of patients with similar distributions of age, AR‐V7 status and CTC counts; which were tested with appropriate two‐sided statistical tests (Mann–Whitney *U* test and Fisher's exact test) where we used *P* > 0.1 to propose no difference.

The outcome of interest was overall survival (OS), as measured from the inclusion of the study until death from any cause. Based on results in our discovery cohort, we calculated that in order to have 80% power to detect a similar HR in the validation cohort given a two‐sided α of 5%, a minimum of 32 events (i.e. deaths) needed to be observed. The included validation cohort (*n* = 50) contained sufficient events.

### 2.2 Enrichment of CTC

As previously detailed by Isebia *et al*. [1] and Onstenk *et al*. [17] for the CABA‐V7 and CABARESC trials, enrichment and the subsequent enumeration of CTCs were performed similarly. Briefly, blood samples were collected in CellSave Preservative tubes (CS) (Menarini Silicon Biosystems, Bologna, Italy) and processed within 96 h after withdrawal using the CellSearch^®^ system (Menarini Silicon Biosystems) according to the manufacturer's instructions. Briefly, CTC enumeration was performed on 7.5 mL of CS blood using the Circulating Epithelial Cell Kit on the CellSearch system. Using ferrofluid labelled anti‐EpCAM antibodies, immunomagnetically captured cells are characterized using different combinations of staining reagents.

### 2.3 Blood sampling and cfDNA extraction

Per included patient (CABA‐V7 and CABARESC), collected blood was centrifuged twice to obtain plasma, as detailed by van Dessel *et al*. [18], and plasma was stored. Plasma from an additional 10 mL EDTA or CS tube was isolated by two centrifugation steps of 10 min at room temperature, at 1711 and 12 000 ***g***, respectively. EDTA tubes were processed within 24 h and CS tubes within 96 h, respectively. Plasma was then immediately stored at −80 C in 2 mL aliquots until cfDNA isolation. Next, cfDNA was isolated using the QIAamp^®^ Circulating Nucleic Acid kit (QIAGEN, Venlo, The Netherlands) and Maxwell^®^ RSC ccfDNA Plasma Kit (Promega, Madison, WI, USA) according to the manufacturer's instructions.

### 2.4 mFAST‐SeqS, sequencing and data analysis

We employed the mFast‐SeqS platform on 1 ng of cfDNA to obtain low‐resolution copy‐number profiles. Aneuploidy scores per chromosome arm were determined using the mFast‐SeqS method. The original Fast‐SeqS [4] was developed as an alternative for the Noninvasive Prenatal Test (NIPT), which screens foetal aneuploidy from maternal blood, and was adapted by Belic *et al*. [8] to estimate tumour fractions in cfDNA using a genome‐wide *Z*‐score that serves as an estimate for overall aneuploidy. As described by Belic *et al*. [8], we used target‐specific LINE‐1 primers and performed a primary PCR step with Phusion Hot Start II Polymerase II to amplify LINE‐1 amplicons. A secondary PCR is performed in which sequencing adaptors and sample‐specific indexes are added to the LINE‐1 amplicons. After each PCR, the amplicons were purified with 1.4× AMPure XP beads (Beckman Coulter, Brea, CA, USA), as described by the manufacturer. The LINE‐1 libraries are quantified with the NEBNext Library Quantification Kit for Illumina (New England Biolabs, Ipswich, MA, USA). Per sequence run, 24 samples were pooled equimolarly (2 nm) and supplemented with a 5% PhiX control library. The libraries were sequenced single‐ended for 150 bp reads on a MiSeq‐sequencer (Illumina, San Diego, CA, USA). Samples yielding < 90 000 reads were sequenced again, and resulting reads were combined [19].

Primer sequences were trimmed from the sequenced reads using trimmomatic [7] (v0.38) prior to alignment on the human reference genome hg19 using Burrows‐Wheeler alignment [8] (v0.7.17). Reads with mapping qualities < 15 were excluded; remaining reads were used to obtain a total read count per chromosomal arm. The short arms of the acrocentric chromosomes 13p, 14p, 15p, 22p and chromosome Y contain too few LINE‐1 elements for proper analysis and were therefore excluded from analysis. Per chromosomal arm, a *Z*‐score (measure for deviation from a reference panel of healthy/diploid male subjects (*n* = 17) from Belic *et al*.) [8] was calculated by subtracting the mean and dividing by the standard deviation of normalized read‐counts for the respective chromosome arm to assess over‐ and under‐representation [8]. *Z*‐scores per chromosome arm were squared and summed into a genome‐wide aneuploidy score per patient. Samples with a genome‐wide *Z*‐score ≥ 5 (a threshold originally set by Belic *et al*. [8]) were considered aneuploid, indicating the presence of ctDNA within the total pool of cfDNA.

### 2.5 Association of aneuploidy scores with other clinical and molecular characteristics

Using the maximum variant allele frequencies per sample as derived from our targeted QIASeq panel as detailed by Isebia *et al*. [15], we performed a two‐sided Spearman's rank correlation coefficient (ρ) versus aneuploidy scores to determine possible trends. In addition, we also performed this between aneuploidy scores and CTC counts at baseline (7.5 mL). A linear model (x ~ y) was also constructed to highlight potential monotonic relationships.

### 2.6 Generating an alternative mFast‐SeqS dichotomization threshold for the stratification of overall survival

We utilized the cutpoint r package (version 1.1.2) [20] to establish an alternative threshold for the dichotomization of the mFast‐SeqS scores by maximizing the sensitivity and specificity for stratifying our patients from the discovery cohort (*n* = 131) based on their survival status (dead vs. alive); using 10.000 bootstrap iterations.

### 2.7 Statistical analysis of overall survival

The most relevant baseline characteristics from the discovery cohort (*n* = 131) for predicting OS, as measured from inclusion of study until death from any cause, were studied in a univariate Cox proportional hazards regression analysis. The following characteristics were investigated: age at registration, total Gleason‐score, PSA at primary diagnosis, dichotomized WHO‐status (0 vs. 1–2), albumin levels (ALB), absolute neutrophil count (ANC), haemoglobin levels (HBG), number of white blood cells (WBC), alkaline phosphatase levels (ALP), lactate dehydrogenase levels (LDH), dichotomized aneuploidy scores at baseline (aneuploidy score < 5 vs. ≥ 5) and dichotomized number of CTCs (per 7.5 mL) at baseline (< 5 vs. ≥ 5 CTCs). We only retained characteristics with *P* < 0.1 for subsequent multivariable Cox proportional hazards regression where backward selection was applied with a threshold of *P* < 0.1. In addition, sample‐specific dichotomized aneuploidy scores and dichotomized CTC counts were combined to identify potential complimentary effects. Kaplan–Meier curves were generated for dichotomized aneuploidy score, dichotomized CTC count and dichotomized WHO status and obtained curves were tested using the log‐rank test.

All analyses were performed on the statistical language platform r (version 4.2.1).

## 3 Results

### 3.1 Baseline characteristics of the discovery and validation cohort

**Fig. 1:**
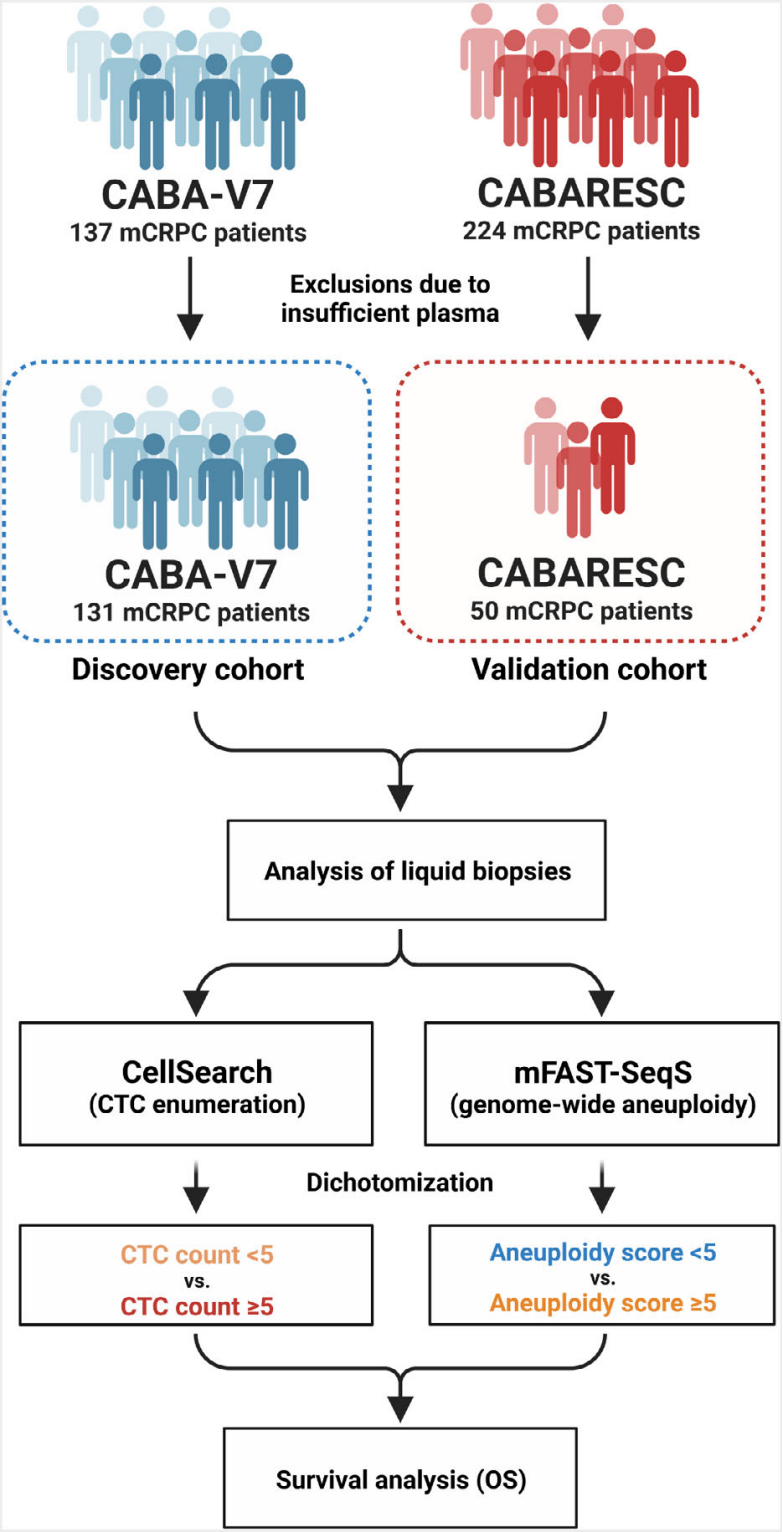
Schematic overview of the included mCRPC cohorts and workflow. For both the discovery (CABA‐V7; *n* = 131) and validation (CABARESC; *n* = 50) cohorts, CTC counts and genome‐wide aneuploidy scores (mFast‐SeqS assay) were derived to determine their prognostic potential on overall survival (OS) of mCRPC patients.

All mCRPC patients from the CABA‐V7 study with successful measurements of both CTC counts and aneuploidy scores served as the discovery cohort (*n* = 131; 92 deaths; Fig. 1). Additionally, we selected a comparable subset of 50 patients (41 deaths) from the CABARESC study for use as a comparable validation cohort (Fig. 1; Table S1). Baseline characteristics of both included cohorts are described in Table 1. No significant differences in baseline characteristics were observed between these cohorts. In addition, no significant differences in clinical characteristics were found between the included validation set (*n* = 50) and the complete CABARESC study group (*n* = 224) from which the validation cohort was drawn (Table S1). The median age at registration for the CABA‐V7 and CABARESC cohorts was 70 and 67 years, respectively. All included patients had a WHO performance status < 3, with the majority of patients having a WHO PS of 1. The initial PSA was 376.5 [standard deviation (SD): 930] in the CABA‐V7 trial and 306.8 (SD: 381.6) in the CABARESC trial, subsequently. Per patient, mFast‐SeqS was performed to generate genome‐wide aneuploidy scores (Fig. S1a), which were subsequently dichotomized into two groups: aneuploidy score < 5 or aneuploidy score ≥ 5.

**Table 1:** Baseline characteristics of included patients within the discovery and validation cohorts. PSA, prostate‐specific antigen; range, min. to max.; SD, standard deviation; WHO PS, World Health Organization Performance Score.

Characteristic	CABA‐V7 (*N* = 131)	CABARESC (*N* = 50)	*P*
Age at registration			
Median (range) – years	70 (46–89)	67 (49–82)	0.487^a^
WHO PS at registration – no. (%)			
0	50 (38.2%)	23 (46%)	0.429^b^
1	72 (55%)	27 (54%)	
2	9 (6.8%)	0 (0%)	
Initial PSA, μg·L^−1^			
Mean ± SD	376.5 ± 930	306.8 ± 381.6	0.61^a^
Median (range)	85.6 (0.2–8185)	165 (4.7–2000)	
Absolute neutrophil count			
Mean ± SD	5.4 ± 2.4	5.9 ± 2.5	0.246^a^
Median (range)	4.9 (0.7–11.8)	5.6 (2.1–14)	
Haemoglobin – g·L^−1^			
Mean ± SD	7.7 ± 0.9	7.5 ± 0.8	0.339^a^
Median (range)	7.7 (4.5–10.3)	7.7 (5.5–9.1)	
Alkaline phosphatase – IU·L^−1^			
Mean ± SD	167.6 ± 171.5	198 ± 164.2	0.287^a^
Median (range)	121.5 (42–1608)	143.5 (50–869)	
Lactate dehydrogenase – IU·L^−1^			
Mean ± SD	335.5 ± 299	387.6 ± 290.6	0.297^a^
Median (range)	238.5 (52–1770)	316 (160–1843)	

a Mann–Whitney *U* test.

b Chi‐square test.

### 3.2 Prognostic value of dichotomized CTC counts and aneuploidy scores

**Fig. 2:**
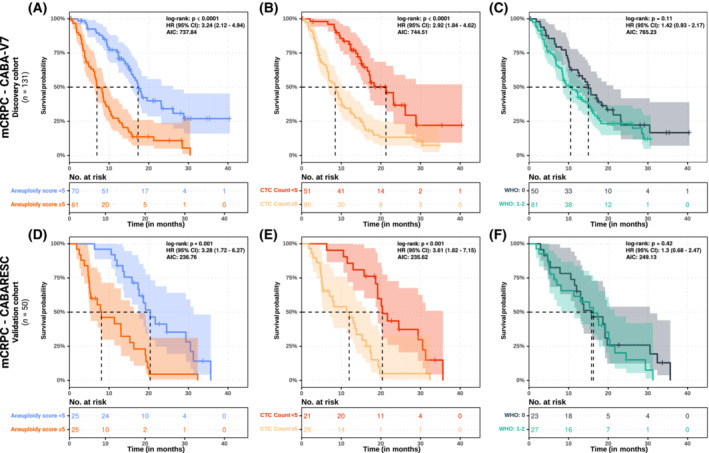
Overall survival versus dichotomized aneuploidy scores, dichotomized CTC counts and WHO status. Survival probability (OS), as measured from inclusion in the study until death from any cause, using univariate analysis of all patients per cohort (*y*‐axis), stratified and coloured by varying dichotomized categories at baseline, is depicted in months (*x*‐axis); censoring is shown by crosses (+). The bottom table represents the total number of remaining cases per depicted time‐point. The log‐rank *P*‐value, hazard ratio (death) with 95% CI and Akaike information criterion (AIC) is shown on the right‐hand top side. The 50% survival probabilities per strata are indicated by dashed lines, whilst the confidence interval per stratum is indicated by transparent backgrounds. (A–C) dichotomized aneuploidy scores, CTC counts and WHO status on the discovery cohort, (D–F) dichotomized aneuploidy scores, CTC counts and WHO status on the validation cohort.

**Fig. 3:**
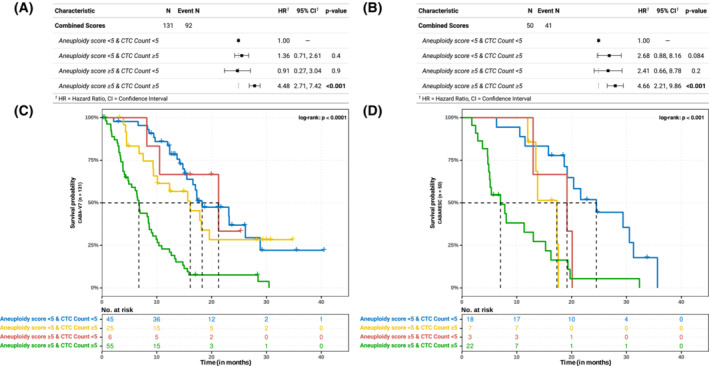
The combined effect of dichotomized CTCs and genome‐wide *Z*‐scores on OS. Survival probability (OS) using univariate analysis of all included patients per cohort (*y*‐axis), stratified and coloured by combined dichotomized CTC counts and genome‐wide *Z*‐score categories, depicted in months (*x*‐axis); censoring is shown by crosses (+). The bottom table represents the total number of remaining cases per depicted time‐point. The log‐rank *P*‐value (between all groups) is shown on the right‐hand top‐side. (A) Cox proportional hazards regression of the combined scores for the discovery cohort; (B) same as (A) but for the validation cohort; (C) Kaplan–Meier curves of the combined scores for the discovery cohort; (D) same as (C) but for the validation cohort.

In the discovery cohort, WHO dichotomized aneuploidy scores (aneuploidy score < 5 or aneuploidy score ≥ 5) and dichotomized CTC counts (< 5 or ≥ 5) were analysed for predicting OS using Cox proportional hazards regression analysis including all relevant clinical characteristics (based on backward selection). This revealed that dichotomized aneuploidy scores and dichotomized CTC counts both independently served as significant non‐invasive biomarkers predicting OS (*q* < 0.001 and *q* = 0.01, respectively; Table 2). Within our discovery cohort, hazard ratios (HR) and confidence intervals (CI) for dichotomized aneuploidy scores and dichotomized CTC counts were respectively 3.24 (2.12–4.94) and 2.92 (1.84–4.62); within our validation cohort, these were 3.28 (1.72–6.27) and 3.61 (1.82–7.15), respectively. To illustrate the prognostic potential of these non‐invasive markers, Kaplan–Meier curves for these characteristics and WHO status are shown for both our discovery and validation cohorts (Fig. 2). Assessment of the potential complementary benefit of both minimally‐invasive markers revealed that patients with both ≥ 5 CTCs and aneuploidy score ≥ 5 could be considered as the group with the worst prognosis compared to CTC < 5 and aneuploidy score < 5 in both cohorts (HR of 4.48 and 4.66 in the discovery and validation cohorts, respectively; Fig. 3). In addition, we observed potential correlations between aneuploidy scores and maximum variant allele frequencies (ρ = 0.47 and *P* < 0.001, Fig. S1b) and between aneuploidy scores and CTC counts (ρ = 0.67 and *P* < 0.001, Fig. S1c).

**Table 2:** Multivariate OS analysis on the discovery cohort. CI, confidence interval; HR, hazard ratio. Hazard ratios with 95% confidence interval (CI) from multivariate Cox proportional hazards regression within the discovery cohort (*n* = 131). The *P*‐values for each multivariate assessment are presented on the right‐hand side of each comparison.

Characteristic	*N*	Event *N*	HR	95% CI	*P*‐value
Genome‐wide status (Baseline)					
Aneuploidy score < 5	131	92	1.00	–	**< 0.001**
Aneuploidy score ≥ 5			2.49	1.57, 3.97	
Dichotomized CTC count (baseline)					
CTC Count < 5	131	92	1.00	–	**0.007**
CTC Count ≥ 5			1.98	1.20, 3.29	
WHO status (pooled)					
0	131	92	1.00	–	0.078
1–2			1.46	0.95, 2.24	

Bold indicates statistical significant value (*P* < 0.05).

Our previous threshold for the dichotomization of mFast‐SeqS scores (< 5 or ≥ 5) was based on previous studies [10, 11, 21]. We also determine a possible alternative threshold for this dichotomization. By maximizing the stratification of survival status within our discovery cohort, we observed a possible alternative threshold (< 1.922707406 or ≥ 1.922707406), which captured a slightly higher HR of 3.79 (2.4–5.99) and lower AIC (731.36 vs. 737.84) within the discovery cohort compared to the other literate‐based threshold (Fig. S2a). This alternative threshold was also validated within the CABARESC cohort, which yielded significant results yet with a lower HR of 2.46 (1.28–4.73) and a higher AIC (242.24 vs. 236.76) compared to the literature‐based threshold (Fig. S2b).

## 4 Discussion

Numerous efforts and studies have been undertaken to study and improve the prognostic value of non‐invasively derived markers in mCRPC patients [2, 3, 4, 5, 6, 22, 23, 24, 25]. From this, ctDNA and CTCs have emerged as some of the most promising biomarkers with prognostic applicability in prostate cancer patients.

Different methods exist for detecting ctDNA within the total pool of cfDNA. These ctDNA analyses are variable in their design; some focusing on single tumour‐specific gene alterations, whilst others, for example, quantify ctDNA by utilizing aneuploidy detection based on copy number alterations [1] or single nucleotide polymorphism (SNP) [26]. In addition, CTC counts can also serve as prognostic markers in mCRPC patients. Within the CARD trial, baseline CTC counts were shown to be prognostic, and de Bono *et al*. [5] showed that dichotomized CTC count is an independent predictor of OS in mCRPC patients.

The exploitation of dichotomized aneuploidy scores as prognostic markers for mCRPC patients has not yet been investigated, whilst it could complement CTC enumeration. In this analysis, we investigated the prognostic value of aneuploidy scores for mCRPC patients and compared their performance to dichotomized CTC counts.

We observed a prognostic value for OS for cfDNA‐based dichotomized aneuploidy scores. These findings were significant in our discovery cohort (CABA‐V7) and were replicated in our comparable mCRPC validation cohort (CABARESC). Dichotomized aneuploidy scores and their combination with dichotomized CTC counts hold complementary benefits in identifying the mCRPC patients with the worst outcome. Additionally, we revealed that dichotomized aneuploidy scores and dichotomized CTC counts both independently serve as significant non‐invasive biomarkers for predicting OS.

Despite these significant observations, this work has limitations. Our employed thresholds for dichotomization of CTC counts and aneuploidy scores are currently based on the previously employed threshold [5, 8], which was found to hold prognostic value. However, we also noted significant predictions of OS when fluctuating these dichotomization thresholds and/or analysing these measurements as continuous variables rather than as a dichotomized variables. This suggests that other potential thresholds or read‐outs could be employed when utilizing these measurements to predict OS. Overall survival was defined as the time from inclusion in the study until death from any cause, rather than cancer‐related death. For our validation cohort (CABARESC), we selected a subset of the initially enrolled CABARESC trial patients. Due to limitations on the availability of remaining plasma, only 50 patients were selected as the validation cohort. Despite this selection, we showed that no significant differences between the full CABARESC cohort and this subset were observed. Furthermore, we ensured a selection of patients with similar distributions of age, AR‐V7 status, CTC counts and overall survival compared to the full cohort (Fig. S3). In addition, the current threshold (< 5 or ≥ 5) used in the dichotomization of aneuploidy status is based on previous literature. This threshold could be scrutinized further in order to optimize the stratification of patients (e.g., overall survival). This should be performed in a larger pan‐cancer cohort whilst taking care not to overfit.

## 5 Conclusion

In conclusion, the mFast‐SeqS‐derived aneuploidy score is a global, minimally invasive and user‐friendly assay that can be employed unrestrictedly without the use of specialized equipment such as CellSearch. In this study, we showed that a dichotomized aneuploidy score is a clinically relevant stratification marker, akin to an established CTC count. The mFast‐SeqS method can be used for an estimation of ctDNA percentage, since it correlates with tumour content and can be easily used for monitoring disease progression without prior knowledge of the genetic composition of the malignancy. The mFAST‐SeqS assay provides an intuitive low‐resolution copy number profile, requiring only a low input of 1 ng cfDNA and results can typically be obtained within 2 days. Therefore, this affordable assay represents an attractive stand‐alone stratification tool for usage in daily clinical practice, which could also aid as a stratification factor in clinical studies. Whether mFast‐SeqS‐derived aneuploidy score could also serve as a predictive biomarker for therapy response or longitudinal therapy monitoring should be further investigated.

## Conflict of interest

All authors certify that all conflicts of interest, including specific financial interests and relationships and affiliations relevant to the subject matter or materials discussed in the manuscript (e.g., employment/affiliation, grants or funding, consultancies, honoraria, stock ownership or options, expert testimony, royalties, or patents filed, received, or pending), are the following:

- Ronald de Wit has acted in a consulting or advisory role for Sanofi, Merck, and Bayer and has received research funding from Bayer, Sanofi.
- Martijn P. Lolkema has acted in a consulting or advisory role for Sanofi, Johnson & Johnson, Merck, Astellas, Incyte, Amgen, Janssen Cilag, Bayer, Servier and Pfizer; and has received research funding from Sanofi, Astellas, Janssen Cilag and MSD.
- John. W. M. Martens has acted in a consulting or advisory role for Novartis and Roche; and has received research funding from Philips, Therawis, Pamgene, Cergentis, Cytotrack.
- Bianca Mostert has acted in a consulting or advisory role for Servier, Lilly and BMS; and has received research funding from Sanofi, Pfizer and BMS.
- Ron H.J. Mathijssen received research funding from Astellas, Bayer, Cristal Therapeutics, Novartis, Pamgene, Pfizer, Roche, Sanofi, Boehringer‐Ingelheim and Servier.

The remaining authors have nothing to disclose.

## Author contributions

KTI, JvR, JWMM and MPL had full access to all the data in the study and take responsibility for the integrity of the data and the accuracy of the data analysis. Study concept and design: MPL, JWMM, BM, SMW, RdW. Acquisition of data: KTI, BM, TD, JK, VdW, EOH, PH, BCMH, HHH, RdW, RHJM, MPL, SMW, JvR, JWMM. Analysis and interpretation of data: KTI, JvR, MPL, SMW, TD, BM, EOH, RHJM, JWMM. Drafting of the manuscript: KTI, JvR, MPL. Critical revision of the manuscript for important intellectual content: KTI, BM, TD, JK, VdW, EOH, PH, BCMH, HHH, RdW, RHJM, MPL, SMW, JvR, JWMM. Statistical analysis: KTI, JvR, EOH. Obtaining funding: MPL, RdW, BM. Administrative, technical, or material support: KTI, JvR, SMW, TD, VdW, JK, BCMH, PH, HHH. Supervision: JWMM.

### Peer review

The peer review history for this article is available at https://www.webofscience.com/api/gateway/wos/peer‐review/10.1002/1878‐0261.13449.

## Supporting information


**Fig. S1.** Association of aneuploidy scores with other molecular characteristics. (a) Aneuploidy scores (y‐axis, signed logarithmic scale) per dichotomized aneuploidy group. Boxplots represent the median and first and third quantile whilst error bars depict the interquartile range (IQR)x1.5. (b) Aneuploidy scores (y‐axis, signed logarithmic scale) versus sample‐specific maximum variant allele frequency derived from a targeted QIASeq panel of 57 genes (x‐axis, signed logarithmic scale). Spearman correlation coefficient (ρ) and statistical significance of observed association (*p*), together with a linear model equation as depicted by a blue line with 95% confidence level interval as gray background, is shown in top. (c) Same a b) but for CTC counts (x‐axis, signed logarithmic scale).

**Fig. S2.** Overall survival versus alternative dichotomized aneuploidy scores. Survival probability (OS), as measured from inclusion of study until death from any cause, using univariate analysis of all patients per cohort (y‐axis), stratified and colored by varying dichotomized categories at baseline, depicted in months (x‐axis); censoring is shown by crosses (+). The bottom table represents the total number of remaining cases per depicted time‐point. The log‐rank *p*‐value, hazard ratio (death) with 95% CI and Akaike information criterion (AIC) is shown on the right‐hand top‐side. The 50% survival probabilities per strata as indicated by dashed lines whilst the confidence interval per stratum is indicated by transparent backgrounds. a) Discovery cohort (CABA‐V7), b) Validation cohort (CABARESC).

**Fig. S3.** Overall survival for all included patients in the discovery cohort, full CABARESC cohort and the included CABARESC cohort as validation cohort. Survival probability (OS) using univariate analysis of all included patients per cohort (y‐axis), stratified and colored by cohort, depicted in months (x‐axis); censoring is shown by crosses (+). The bottom table represents the total number of remaining cases per depicted time‐point. Hazard ratios with 95% confidence interval (CI) from multivariate Cox proportional hazards regression within the discovery cohort (*n* = 131). The *p*‐values for each multivariate assessment are presented on the right‐hand side of each comparison.

**Table S1.** Baseline characteristics of the complete CABARESC cohort and the included subset used for validation.

**Table S2.** Overview of included patients and data presented in figures. Overview of all data as presented and quantified in this manuscript.

## Data Availability

All supporting data of this study as used within the analysis and as‐presented within the figures are available in the supplementary material (Table S2) of this article. All utilized custom code and scripts used throughout this manuscript are deposited at https://doi.org/10.5281/zenodo.7801894.
