# Supplementary figures and images for: mFast‐SeqS‐based aneuploidy score in circulating cell‐free DNA is a prognostic biomarker in prostate cancer
Source: Mol Oncol. 2023 Aug 18;17(9):1898–907. doi: 10.1002/1878-0261.13449 (PMC10483599; doi:10.1002/1878-0261.13449)

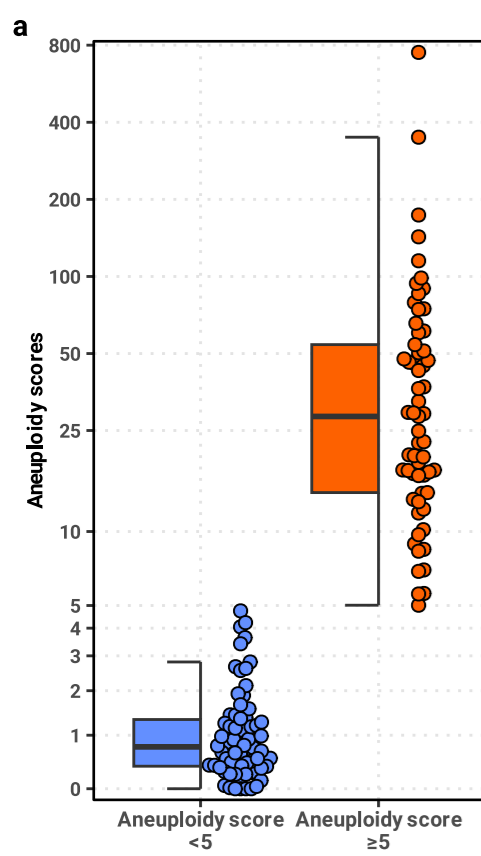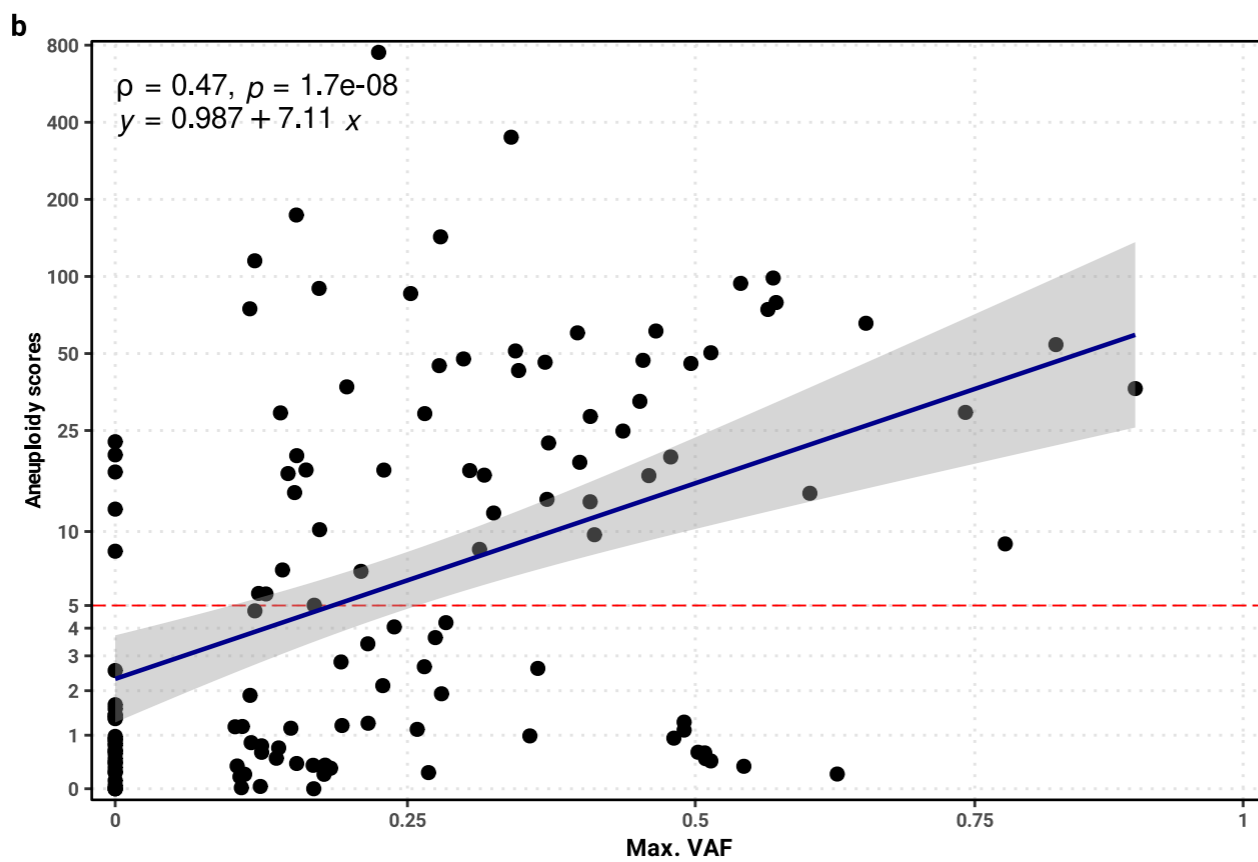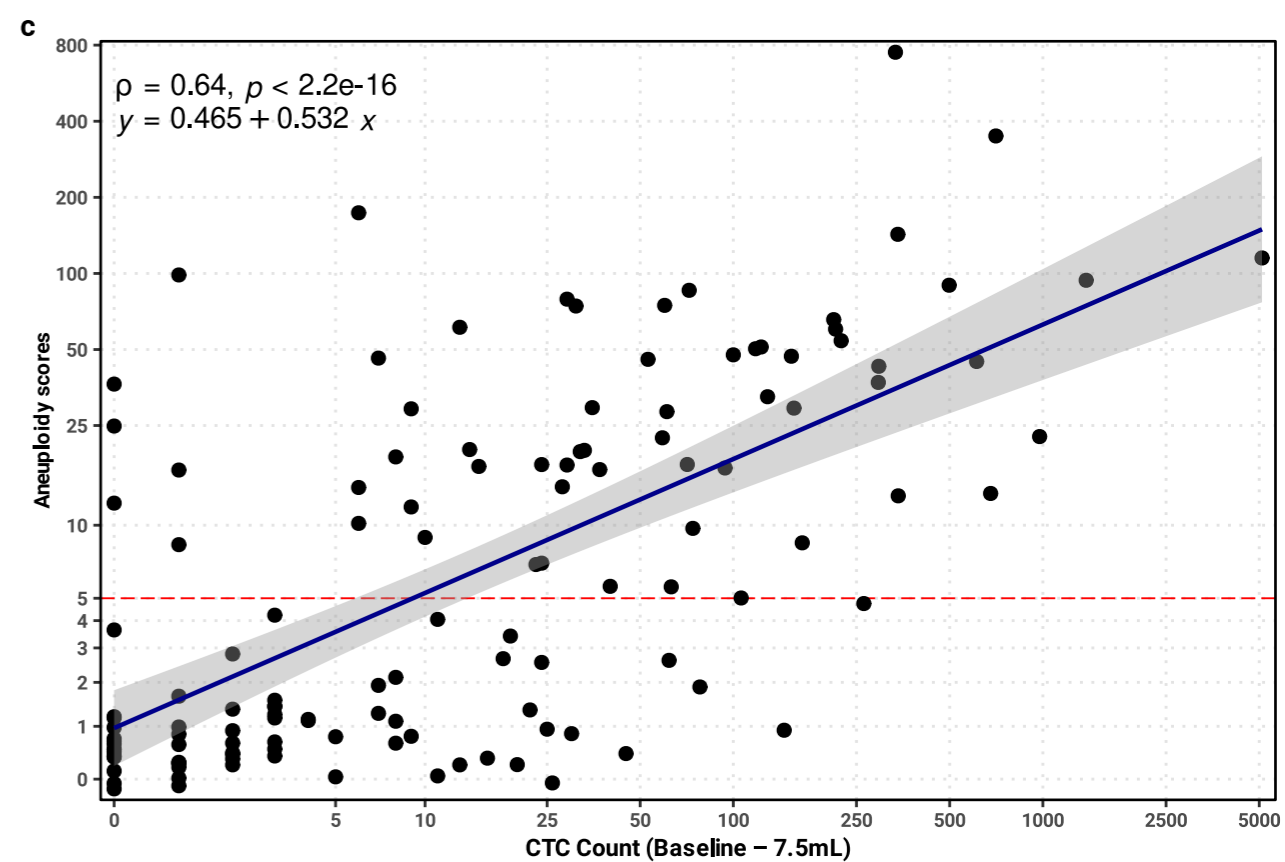

Supplement: Supplementary file 1 — Fig. S1. Association of aneuploidy scores with other molecular characteristics. (a) Aneuploidy scores (y‐axis, signed logarithmic scale) per dichotomized aneuploidy group. Boxplots represent the median and first and third quantile whilst error bars depict the interquartile range (IQR)x1.5. (b) Aneuploidy scores (y‐axis, signed logarithmic scale) versus sample‐specific maximum variant allele frequency derived from a targeted QIASeq panel of 57 genes (x‐axis, signed logarithmic scale). Spearman correlation coefficient (ρ) and statistical significance of observed association (p), together with a linear model equation as depicted by a blue line with 95% confidence level interval as gray background, is shown in top. (c) Same a b) but for CTC counts (x‐axis, signed logarithmic scale). [file MOL2-17-1898-s005.pdf]

a

mCRPC- CABA-V7

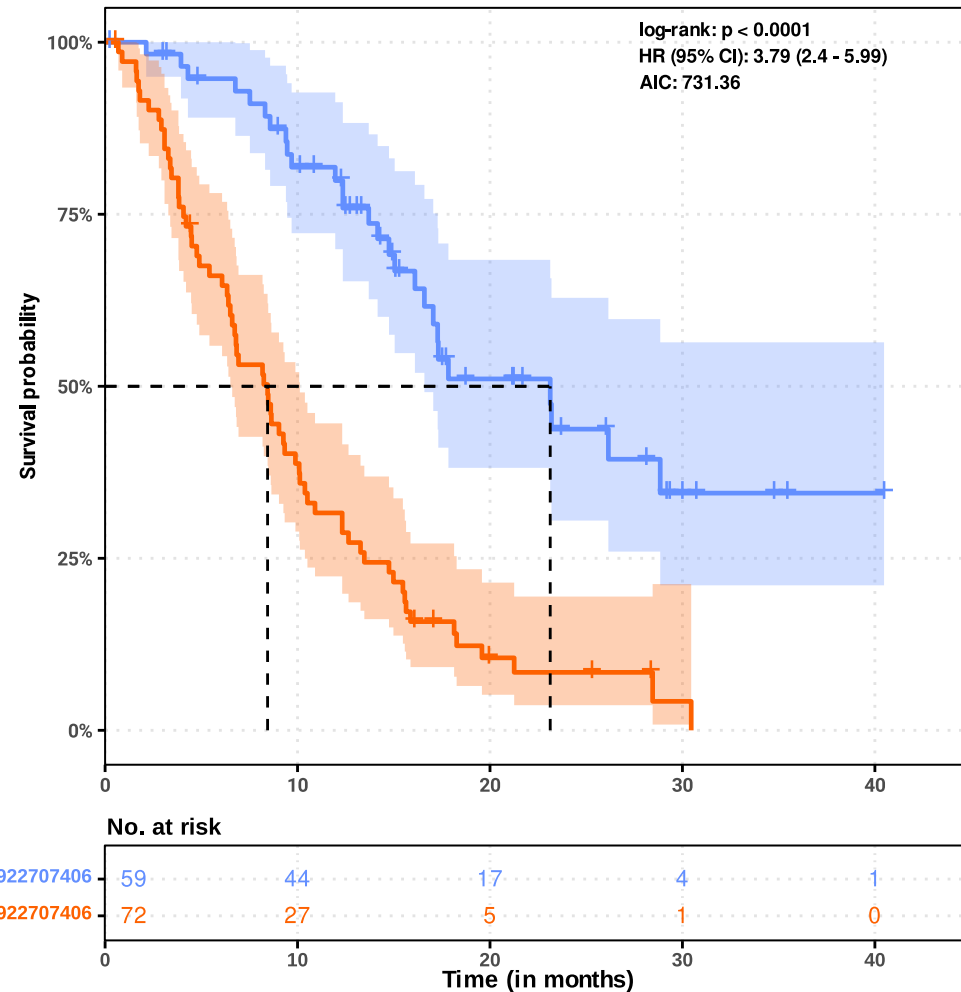

b

mCRPC - CABARESC

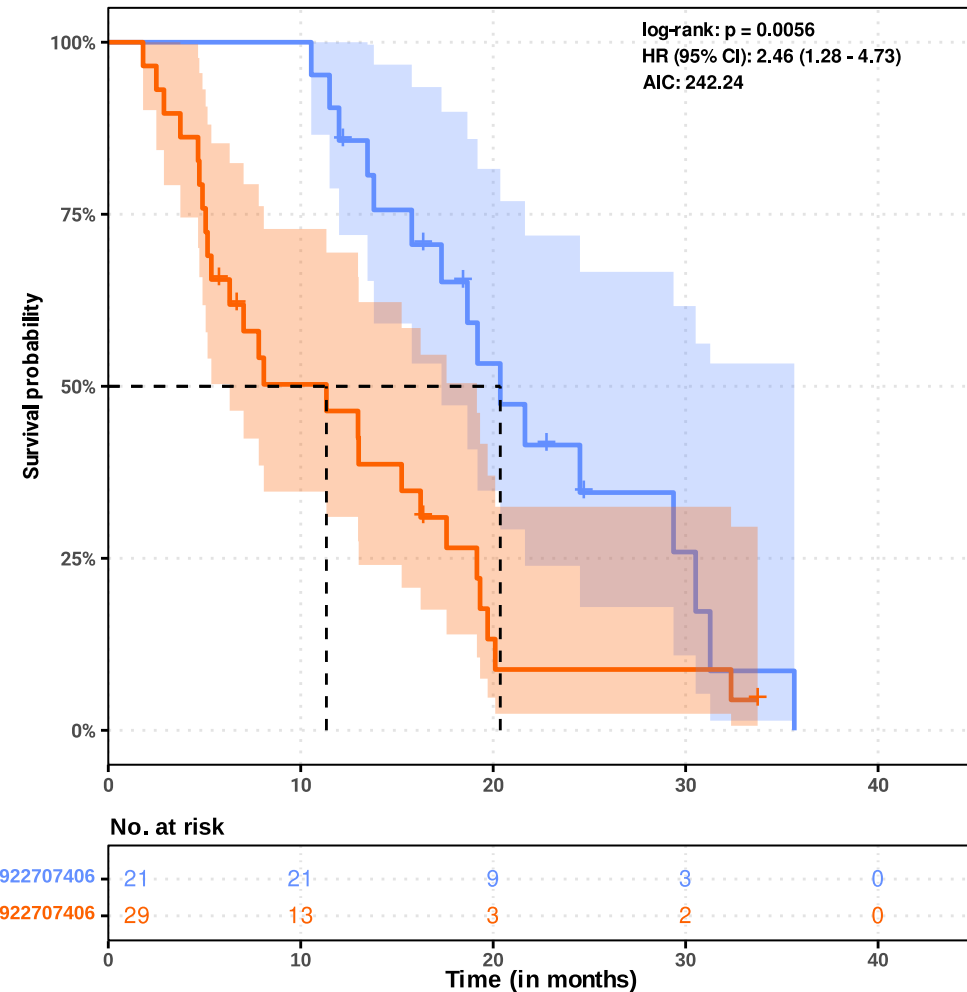

Supplement: Supplementary file 2 — Fig. S2. Overall survival versus alternative dichotomized aneuploidy scores. Survival probability (OS), as measured from inclusion of study until death from any cause, using univariate analysis of all patients per cohort (y‐axis), stratified and colored by varying dichotomized categories at baseline, depicted in months (x‐axis); censoring is shown by crosses (+). The bottom table represents the total number of remaining cases per depicted time‐point. The log‐rank p‐value, hazard ratio (death) with 95% CI and Akaike information criterion (AIC) is shown on the right‐hand top‐side. The 50% survival probabilities per strata as indicated by dashed lines whilst the confidence interval per stratum is indicated by transparent backgrounds. a) Discovery cohort (CABA‐V7), b) Validation cohort (CABARESC). [file MOL2-17-1898-s004.pdf]
