# Supplementary material for: mFast‐SeqS‐based aneuploidy score in circulating cell‐free DNA is a prognostic biomarker in prostate cancer
Source: Mol Oncol. 2023 Aug 18;17(9):1898–907. doi: 10.1002/1878-0261.13449 (PMC10483599; doi:10.1002/1878-0261.13449)
Supplement: Supplementary file 3 — Fig. S3. Overall survival for all included patients in the discovery cohort, full CABARESC cohort and the included CABARESC cohort as validation cohort. Survival probability (OS) using univariate analysis of all included patients per cohort (y‐axis), stratified and colored by cohort, depicted in months (x‐axis); censoring is shown by crosses (+). The bottom table represents the total number of remaining cases per depicted time‐point. Hazard ratios with 95% confidence interval (CI) from multivariate Cox proportional hazards regression within the discovery cohort (n = 131). The p‐values for each multivariate assessment are presented on the right‐hand side of each comparison. [file MOL2-17-1898-s003.pdf]

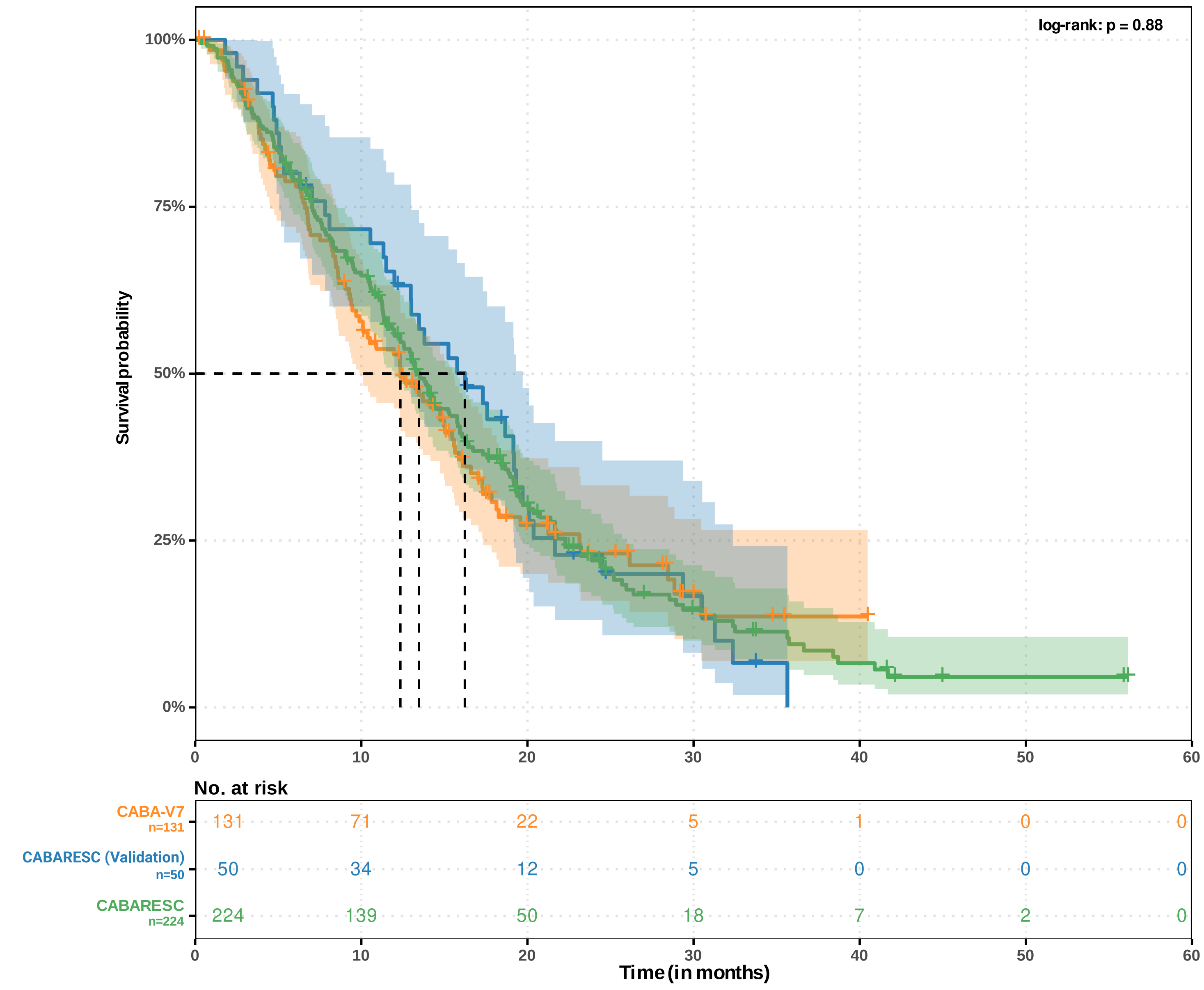
log-rank: p = 0.88
Survival probability
100%
75%
50%
25%
0%
0
10
20
30
40
50
60
No. at risk
CABA-V7
n=131
131
71
22
5
1
0
0
CABARESC (Validation)
n=50
50
34
12
5
0
0
0
CABARESC
n=224
224
139
50
18
7
2
0
Time (in months)
